# Identifying patient data that should be available in community pharmacies for statutory dispensing and providing clinical pharmacy services: A Delphi study

**DOI:** 10.1016/j.rcsop.2026.100716

**Published:** 2026-02-07

**Authors:** Johanna Laakso, Sonja Kallio, Marja Airaksinen, Maarit Dimitrow

**Affiliations:** a Clinical Pharmacy Group, Division of Pharmacology and Pharmacotherapy, Faculty of Pharmacy, University of Helsinki, Viikinkaari 5 E, P.O. Box 56, 00014, Finland; b The Association of Finnish Pharmacies, Elimäenkatu 5, 00510 Helsinki, Finland

**Keywords:** Community pharmacy, Patient data, Dispensing, Medication counseling, Medication safety, Rational pharmacotherapy, Therapeutic outcome monitoring

## Abstract

**Background:**

Community pharmacy practice has evolved from dispensing and medication counseling towards clinical pharmacy services such as medication reviews to ensure rational pharmacotherapy. These functions require better access to patient data than are currently available in community pharmacies. This study aimed to identify and prioritize patient data that should be available in Finnish community pharmacies for 1) statutory dispensing, including medication counseling, and 2) clinical pharmacy services promoting rational pharmacotherapy.

**Materials and methods:**

This study applied a three-round Delphi survey with an expert panel of 20 clinical pharmacists. A consensus was formed using a preliminary list of patient data (39 items) important for managing major long-term diseases and related pharmacotherapies in outpatient care. The list was based on literature and research group's expertise. A consensus ≥80% was required. The responses were analyzed quantitatively and qualitatively.

**Results:**

Most panelists (*n* = 15/19) perceived the current patient data available in community pharmacies insufficient. More patient data should be available, especially for providing clinical pharmacy services, but also for dispensing and related medication counseling (34 vs. 11 items reached a consensus, respectively). For both purposes, patient data on diagnoses, kidney function, and blood pressure were rated most important to be available. Panelists expressed some concerns about community pharmacists' resources and competence to use the data.

**Conclusion:**

Consensus was reached for a relatively large set of patient data items to be available in community pharmacies, especially for providing clinical pharmacy services.

## Highlights

• Community pharmacies should have extended access to electronic patient data.
• Most important patient data were diagnoses, kidney function, and blood pressure.
• Pharmacists' competence to interpret patient data must be ensured.

## 1 Introduction

Community pharmacy practice has evolved globally from dispensing towards patient-care-oriented services ensuring rational, i.e., effective, safe, high quality, cost-effective and equal, pharmacotherapy.1., 2, 3, 4, 5. In addition to dispensing and related medication counseling, pharmacies provide services to prospectively prevent medication-related problems and risks to ensure optimum outcomes of pharmacotherapies. Community pharmacists' involvement in promoting rational pharmacotherapy is critically important because majority of medicines are used in outpatient care.^1^ Community pharmacists are among the most accessible healthcare professionals, regularly interacting with outpatients while dispensing their medicines. Therefore, it is essential to integrate the development of community pharmacy services with development of other health services and systems.^6^, ^7^ The most urgent need for integration relates to managing medications of patients with chronic diseases and polypharmacy, as they are at the highest risk of medication-related problems and errors.8., 9., 10

One of the barriers to extending community pharmacists' involvement in patient-centered care is that they have limited independent access to patient data such as laboratory data.^11^, ^12^ Based on international literature, only a few studies have been conducted on community pharmacists' independent access to patient data.^11^, ^12^, ^13^, ^14^ Even less research evidence was found on patient data items that should be available in community pharmacies.^15^, ^16^, ^17^, ^18^ The results of these studies are confirmed by a recently published scoping review that explored barriers to integrating community pharmacists in primary healthcare.^12^

The studies found examined the following aspects of laboratory data use in pharmacy practice: (1) the use of laboratory tests in clinical risk management of potential drug–drug interactions,^15^ (2) the integration of electronic health records into community pharmacy practice,^16^ (3) community pharmacists' access to patient health records for identifying drug therapy problems,^17^ and (4) the usability of laboratory data linked to prescriptions for outpatients.^18^ These studies on integration of patient and prescription data in community pharmacies^13^, ^15^, ^16^, ^17^, ^18^ have found that access to laboratory data in community pharmacies can prevent medication errors and enable community pharmacists to be more involved in supporting patient care. In particular, the study by Yokoyama et al.^18^ in Japan showed that the quality of prescription reviews and efficacy and safety of pharmacotherapy improved when laboratory data were made available in community pharmacies. By providing community pharmacists with extended access to patient data, cooperation with other healthcare professionals can be improved and made more feasible.1., 5., 6, 7, 19, 20, 21, 22, 23. This could unify patient counseling and medication monitoring practices and improve the quality and availability of extended clinical pharmacy services, such as medication reviews.

Finland is an example of a country where licensed pharmacists working in community pharmacies can access only prescription and medication reimbursement data directly from the electronic health records. For any other patient information, they need to ask the patient or contact the physician or another care team member to access patient data. This study aimed to identify and prioritize what patient data should be available in Finnish community pharmacies for 1) statutory dispensing, including medication counseling, and 2) more extended clinical pharmacy services to promote rational pharmacotherapy.

## 2 Materials and methods

### 2.1 Study context

This study was conducted as part of a larger research program on implementing patient care-oriented practices in Finnish community pharmacies.^24^, 25. In Finland, about 600 privately owned community pharmacies and two university-owned teaching pharmacies supply prescription and non-prescription medicines from over 800 outlets and 200 online pharmacies nationwide.^26^ Pharmacy personnel consist mostly of licensed pharmacists with at least a BSc degree; pharmacy owners must have an MSc (Pharm) degree and a license granted from the Finnish Medicines Agency.

While dispensing, pharmacists are required to review prescriptions for dose, indication, potentially harmful interactions, duplicates, and potential overuse, and to ensure the patient is aware of how to use the medicine safely and appropriately.26., 27. Medication counseling became mandatory in 1983^26^ and since then counseling has been prioritized in strategic service development in community pharmacies, even more systematically since the early 1990s.^28^ As a result of a long-term commitment to counseling services, community pharmacists have become a primary source of medicines information to medicine users together with physicians and statutory package leaflets.28., 29, 30

Finnish community pharmacies also provide extended services like automated dose dispensing (ADD), health screenings, inhalation checks, and medication reviews - many nationally developed and standardized by the Association of Finnish Pharmacies.^24^, 25., 30, 31 Although intended to a large-scale national implementation, many of the services are still provided by a minority of pharmacies, often targeting individual users.^24^, ^31^ Automated dose dispensing (ADD) has nationally extended to geriatric care units in social and primary care and is publicly reimbursed for people over 75 years with polypharmacy.^31^, 32. Other services, such as medication reviews, medication safety audits and personnel training in home care and nursing homes are provided by pharmacies, but they have not yet reached the same kind of standard practice position as ADD.^31^

Pharmacies in Finland have a modern infrastructure for dispensing, counseling, and managing medication risks.^24^, ^33^ They have access to a wide range of electronic databases and portals assisting in reviewing medications, for example, for drug-drug interactions, potentially inappropriate medications (PIMs) for older adults, high-risk medications, and medicine use in renal failure - tools shared across the Finnish healthcare system. With digitalization, multichannel services blending in-person and online options have become common.^34^

### 2.2 Study design and method

**Fig. 1:**
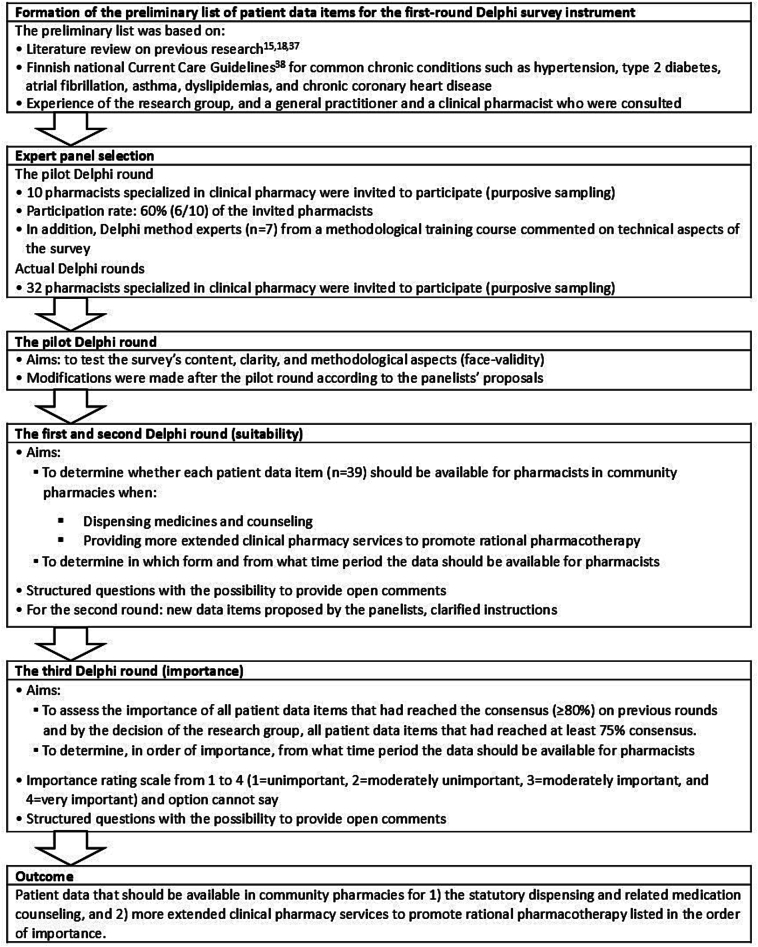
The three-round Delphi survey process.

This study applied a three-round Delphi method (Fig. 1).^35^, ^36^ A Delphi study usually consists of several rounds in which selected expert panel members anonymously and independently provide their judgement of the study subject. Between the rounds the panelists receive aggregated feedback on the results of previous rounds.^36^

### 2.3 Development of the Delphi survey instrument

The Delphi survey instrument was based on a preliminary patient data item list consisting mostly of laboratory tests and other health tests used in the monitoring of common chronic conditions and pharmacotherapies. The list was compiled based on the main author's literature review^37^ on previous research,^15^, ^18^ therapeutic guidelines,^38^ and the expertise of the research group and other experts in the field of the study (Fig. 1). The survey questions aimed to provide information on which patient data items should be available when 1) dispensing medicines and counseling, and 2) providing more extensive clinical pharmacy services promoting rational pharmacotherapy. Before the first Delphi round, the survey instrument was pilot tested with a small group of clinical pharmacy experts (*n* = 6) and Delphi method experts (*n* = 7) who did not participate in the actual Delphi rounds (Fig. 1). The final survey instrument for the first Delphi round comprised 39 patient data items.

### 2.4 Expert panel recruitment

A purposive sample of expert panelists was recruited from pharmacists with theoretical and practical experience in providing clinical pharmacy services, conducting collaborative medication reviews in various healthcare settings, and understanding community pharmacy practices as part of local primary care. Only pharmacists were recruited to ensure that panelists were familiar with the community pharmacy setting and practice, including the process of dispensing medicines and counseling. Panelists were selected to include pharmacists with work experience in community pharmacies and other care settings. This is because pharmacists working in hospitals and health centers have better access to patient data and thus may have a better understanding of how to apply patient data in real-life medication management practice. An invitation to participate in the study was sent to 32 experts. The panel size was decided on the basis of previous evidence of a suitable panel size for this kind of study. A relatively small panel size is found to be effective to provide reliable results if the panelists have a comprehensive understanding in the field of interest.^39^

### 2.5 Delphi rounds

**Fig. 2:**
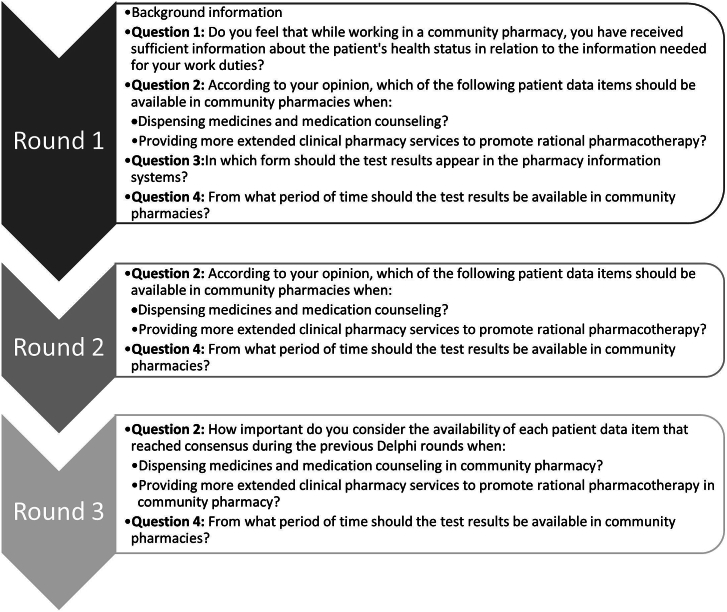
Questions asked to the panelists (*n* = 20) in each Delphi round (*n* = 3).

The actual Delphi rounds were performed online using eDelphi software^40^ between April and June 2022. The duration of each round was two weeks. Between the rounds, the research group had one week to analyze the responses and modify the survey for the next round. The panelists were asked in total four questions during the rounds (Fig. 2). For each question, panelists had structured options for responses and an option for open comments to justify their responses and suggest reformulation of the survey. The first two rounds focused on suitability and the third round on the importance of patient data items. Between the rounds, the main author first analyzed the responses independently. Then the results were discussed in the four-member research group to formulate the next round's survey instrument. Anonymous feedback from the previous round was also available to the panelists, including consensus rates, summary of the responses and open comments. The results were reported applying the Guidance on Conducting and REporting DElphi Studies (CREDES) by Jünger et al.^36^

### 2.6 Data analysis

Patient data items that reached consensus in the first and second rounds were defined as items that should be made available in community pharmacies (Fig. 1). The third round was to assess the importance of each data item that reached consensus in the previous rounds 1 and 2.

All analyses were conducted using Microsoft 365 Excel Spreadsheet Software. Frequencies of the panelists' structured responses were exported from eDelphi software to Excel. In the first and second round, percentages of panelists who agreed that a patient data item should be available in community pharmacies for either dispensing and medication counseling or more extended clinical pharmacy services were calculated. The consensus threshold was set at 80%, which corresponds to the consensus thresholds typically used in Delphi studies.^36^

During the third round, the importance of each patient data item that reached consensus in Rounds 1 and 2 was scored from 1 to 4 (unimportant, moderately unimportant, moderately important, and very important, respectively) by the panelists. From these scores means and standard deviations were calculated and the data items ranked in order of importance to highlight the most important patient data items. The option “cannot say” was excluded from the scoring scale of 1–4 because it could have affected the importance scores. Instead, the number of “cannot say” responses was calculated to assess whether that had an impact on the results.

The main author conducted an initial inductive analysis of the panelists' open comments to identify potential reformulations of the survey questions, recurring thematic patterns, and illustrative quotations from the panelists' responses. Ambiguities in these interpretations were subsequently discussed within the research group to achieve consensus on the finalized reformulations, themes, and selected quotations. Since the comments were primarily intended to guide modifications to the survey for later rounds and the dataset was limited, formal coding procedures and external validation of the thematic analysis were not conducted. Translations to English were independently performed by three authors, followed by comparison and discussion to reach a final agreed version.

### 2.7 Research ethics

This research was conducted according to the national Guidelines for Good Scientific Practice.^41^ Ethics committee pre-evaluation was not required as the study was not invasive medical research involving human participants nor collected any patient-specific health data. Therefore, the study fell outside of the scope of the Medical Research Act (488/1999), which regulates medical research involving humans in Finland.^42^ Participation in the expert panel consisting of health professionals was voluntary. All panelists provided informed consent to participate during the recruitment process. As the study did not involve any physical intervention, exposure to strong stimuli, risk of mental harm, or threat to participant safety, an ethical review statement from a University of Helsinki's human sciences ethics committees was not required.^43^ Panelists' anonymity was ensured, and they were informed about the purpose of the research. Data management throughout the research project was performed according to data protection regulations valid at the study's time.^44^ The study did not require any research permits from health care organizations as the study did not involve patients, personnel, patient data or any other resources from them.

## 3 Results

### 3.1 Expert panelists

Of the 32 experts invited, 20 participated in the study (participation rate 63%). The response rates were 95% (*n* = 19/20) in Round 1, 100% (*n* = 20/20) in Round 2, and 95% (n = 19/20) in Round 3. The panelists (n = 20) were mainly experienced pharmacists with a wide range of work experience in different healthcare settings (Table 1). Most of the panelists (*n* = 18/20) had more than 10 years of work experience, and 12/20 had an MSc (Pharm) degree. All panelists had specialized in clinical pharmacy, most of them were accredited in conducting comprehensive medication reviews (CMRs).

**Table 1:** Characteristics of the pharmacists participating in the expert panel (n = 20).

Degree	n (%)
Bachelor of Science in Pharmacy (a three-year university degree)	7 (35)
Master of Science in Pharmacy (a five-year university degree)	12 (60)
Doctor of Philosophy (PhD) in Pharmacy	1 (5)

Expertise/specialization	
Expertise in medication reviews45., 46., 47, 48	1 (5)
Expertise in comprehensive medication reviews45., 46., 47, 48	14 (70)
Specialization in hospital and health center pharmacy	1 (5)
Other	4 (20)

Work experience as a pharmacist	
1–5 years	1 (5)
6–10 years	1 (5)
More than 10 years	18 (90)

Other degrees in healthcare	
Yes (nurse)	1 (5)
No	19 (95)

Current work setting	
Community pharmacy	6 (30)
Hospital pharmacy	7 (35)
Health center	3 (15)
Other (self-employed pharmacist, primary care, professional association)	4 (20)

Previous full-time work settings (choose one or more)	
Community pharmacy	11 (55)
Hospital pharmacy	4 (20)
Health center	2 (10)
Other	3 (15)
None	3 (15)

### 3.2 Determination of the suitability and importance ratings of the patient data items (Rounds 1–3)

Most of the expert panelists (15/19, 79%) perceived the patient data currently available in community pharmacies as insufficient, while one panelist regarded it as sufficient, and three panelists could not say (Fig. 2: Question 1).

In the first Delphi round, for dispensing and counseling 0/39 data items and for clinical pharmacy services 31/39 data items reached the required consensus rate of 80% in Question 2 (Table 2). However, in their comments regarding dispensing and counseling, the panelists emphasized the significance of numerous individual data items. This pattern suggests that they may have approached the issues primarily from a present-day dispensing-oriented perspective rather than from a broader clinical and prospective risk management standpoint. Thus, it was decided to include all data items in the second round's survey with more detailed instructions highlighting the desired perspectives. For the second round, three new patient data items suggested by the panelists (ferritin, transferrin receptor and albumin) were also added.

**Table 2:**
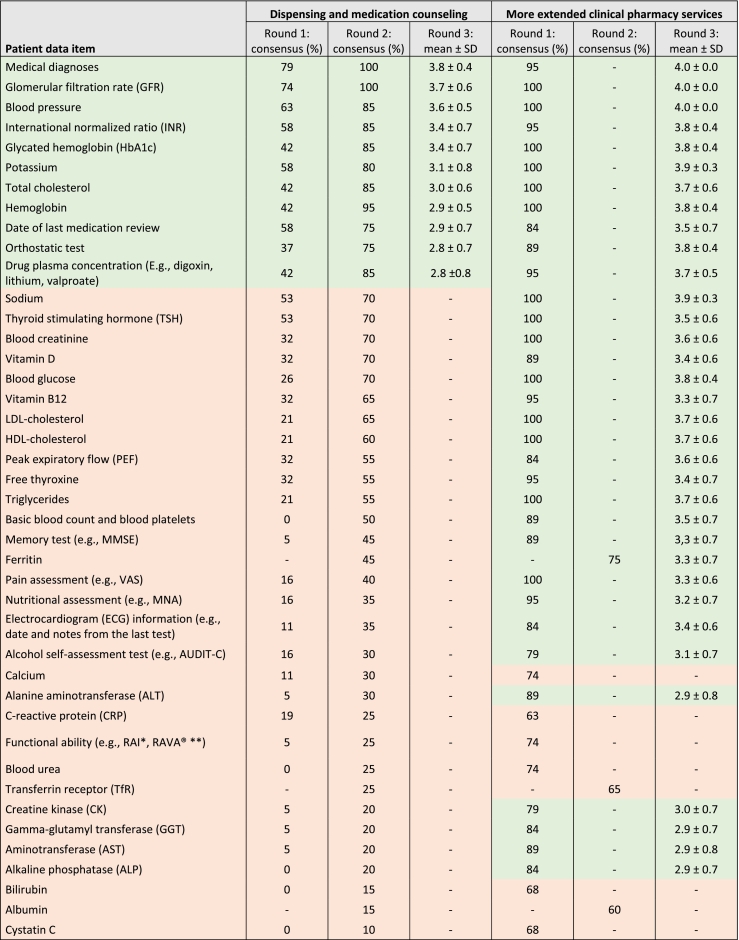
Consensus percentages and importance ratings of each patient data item (*n* = 42) in Rounds 1–3.

All items that reached a consensus (75%) were scored from 1 to 4 (1 = unimportant, 2 = moderately unimportant, 3 = moderately important, and 4 = very important). In addition, there was an option “cannot say” which was not included in the scoring.

* National Institute for Health and Welfare: Resident Assessment Instrument RAI for functional ability^49^

** Finnish Consulting Group (FCG): RAVA Instrument for functional ability^50^^.^

After all three Delphi rounds panelists considered that a more limited set of patient data (9/42 data items) for dispensing and patient counseling would be important to be available for community pharmacists, while a relatively large set of patient data (31/42 data items) would be important for more extended clinical pharmacy services. In addition to the items that had reached consensus in Rounds 1–2, the research group decided to add five items (two items for dispensing and counseling and three for extended services, consensus ≥75%) to the final round to be ranked by the expert panel for importance. Therefore, 11 patient data items for dispensing and counseling and 34 for more extended clinical pharmacy services were rated as important to be available in community pharmacies (Table 2).

**Fig. 3:**
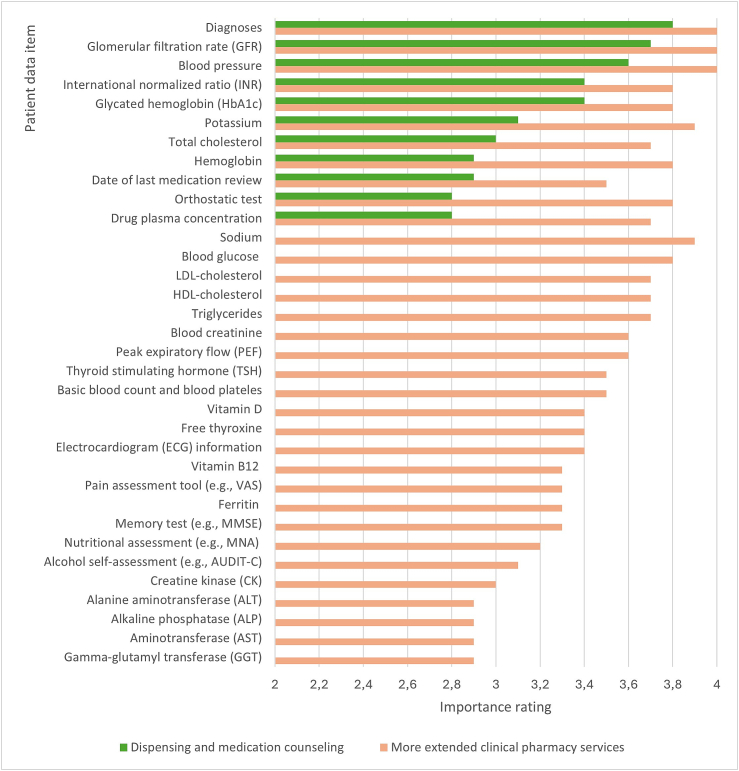
The importance of patient data items to be available in community pharmacies in dispensing and medication counseling (11 items) and in more extended clinical pharmacy services promoting rational pharmacotherapy (34 items). Items were scored from 1 (unimportant) to 4 (very important) according to their importance.

Data about patients' diagnoses, kidney function (GFR) and blood pressure were rated as the most important to be available in community pharmacies (Fig. 3). All data items were scored at least close to moderately important (all scores ≥2.8) during the final round. Only one “cannot say” response was given in the final round.

### 3.3 Format and length of period for keeping the patient data accessible to community pharmacists in patient records

The majority of the panelists (*n* = 17/19, 90%) considered that community pharmacists should have access to the following information about a patient data item: test result, the date when the test was taken, and whether the result was in line with the patient's treatment goals (Fig. 2: Question 3). One of the panelists had the opinion that only the date of the test and whether the test result was in line with the treatment goals should be made available, and one wanted to have available the test result and the date of the test.

Responses to the question about the length of the period from which the test results should be available to community pharmacists varied more (Table 3). During the first two rounds, panelists were asked to choose the most appropriate alternative. As consensus was not reached in these rounds, in the final round, panelists were asked to rank the order of importance for the three alternatives that were most popular in Rounds 1–2 (Table 3). The panelists found the issue to be case- or medicine-specific, but they ranked highest the alternatives “Three latest results” and “One-year history”.

**Table 3:** Panelists' responses to the question “From which time period should the test results be available in community pharmacies?” by each Delphi round.

Alternative	Round 1 n (%)	Round 2 n (%)	Round 3 TOP three alternatives⁎⁎⁎
Only the latest test result	3 (16)	2 (10)	1. Three latest test results (eight panelists rated 1st) 2. 1-year history (eight panelists rated 1st) 3. History as needed (three panelists rated 1st)
Three latest test results⁎	–	6 (30)	
One-year history	11 (58)	7 (35)	
Two-year history	2 (11)	1 (5)	
History as needed⁎	–	4 (20)	
Another alternative, please specify⁎⁎	3 (16)	0 (0)	

⁎ Alternative added to round 2 according to feedback from round 1.

⁎⁎ All proposed alternatives are visible in the table.

⁎⁎⁎ In the final round, panelists were asked to rank the order of importance for the three alternatives that were most popular in Rounds 1–2.

### 3.4 Open comments on the availability of patient data in community pharmacies

Although commenting was voluntary, the panelists commented and justified their responses actively and extensively in each round (Round 1 *n* = 52, Round 2 *n* = 31, Round 3 n = 17). As panelists could provide open comments anonymously on every page of the survey and each panelist could provide multiple or no comments, it was not possible to determine the total number of panelists who commented. To Question 1 relating to panelists' experiences of the sufficiency of patient data in community pharmacies (Fig. 2), panelists commented that currently the only way to obtain patient data is to ask the patient or other healthcare units. The challenge is that patients do not always know their own health measures, and the data they provide are based on their memories and narratives. Requesting patient data from other healthcare units is complicated and time consuming. According to the comments, additional patient data are needed in community pharmacies when conducting medication reviews. Sometimes, it may be necessary to have test results available even when dispensing and counseling (e.g., in cases of drug-drug interactions).

Related to Question 2 regarding patient data items (Fig. 2), authors identified four central themes from the panelists' comments (Table 4) in addition to potential reformulations for the survey. Panelists commented on how patient data should be made available and what challenges may be related to accessing and using the data. Most expressed concerns were related to pharmacists' skills and competence in interpreting patient data and resources in pharmacies. The same themes recurred in each round.

**Table 4:** Themes and examples of panelists' open comments in rounds 1–3 on how patient data should be made available in community pharmacies and what challenges may be related to accessing and using the data (related to Question 2, Fig. 2). Themes were formed using inductive analysis of the open comments.

Theme	Examples of open comments⁎
The extent of patient data items needed in the community pharmacy	“The (patient) data should be available during dispensing to the extent that it is related to the medicine being dispensed, e.g. cholesterol values should be available when dispensing cholesterol medication.” (Round 1) “(The information) does not need to be visible all the time but should be accessible when needed.” (Round 1) “Too much information does not always make work easier and therefore displaying all patient data can complicate its usability.” (Round 1) “In connection with more extended clinical pharmacy services, it would be good to see all laboratory results.” (Round 1)
Benefits of the availability of additional patient data in the community pharmacy	“(Medication) counseling would be easier if you could see the (health) situation (of the patient).” (Round 1) “Blood pressure results are needed especially when dispensing blood pressure medicines and in a more comprehensive medication review. However, blood pressure can elevate, for example, when using anti-inflammatory drugs, so showing the results while dispensing other medicines would improve the safety of pharmacotherapy. The same principle applies to other data items.” (Round 1) “The (patient) data would help providing accurate medication counseling and are very important for treatment monitoring.” (Round 2)
Additional training needs of community pharmacists to have skills to use and interpret patient data	“You also need to know how to interpret these results and understand when and how to possibly address them when dispensing a prescription – training is needed!” (Round 1) “The more (lab) values are visible in the pharmacy during dispensing, the greater the need for competence in interpreting them. In addition to interpreting the values, understanding healthcare processes is essential. If both are lacking, referral of the patient to a physician may not be timely. In order to develop the operation, strong cooperation is required between health centers, hospitals, and community pharmacies, both in terms of training and information exchange.” (Round 1) “An ‘ordinary’ community pharmacist may not necessarily be able to interpret all laboratory values and draw the right conclusions from them.” (Round 2)
Community pharmacy resources to make use of additional patient data	“These (lab) values are important, but in the busy pharmacy setting there is simply no time to review them in detail while dispensing.” (Round 1)

⁎ Quotes were translated from Finnish to English by the authors (see Methods for details).

## 4 Discussion

In this Delphi study, it was evident that in community pharmacies a more limited patient dataset was sufficient for dispensing and counseling than for extended clinical pharmacy services promoting rational pharmacotherapy. However, the study had a clear message that for both purposes more patient data should be made available than is in the current practice in Finland. Data on patients' diagnoses, kidney function (GFR), and blood pressure were rated as the most important to be available.

According to this study, community pharmacists should have access to patient conditions and diagnoses. This opinion is shared by Finnish adult population: in a representative national population survey in 2020 on community pharmacy services, 51% of the respondents (*n* = 1650) perceived that pharmacies should have access to limited health information, and 27% to all health information with the individual's consent.^30^ For customized patient-centered medication counseling, it is essential to know the condition for which the medicine is prescribed, as many medicines have multiple indications. Not knowing the indication may result in conflicting information being provided to the patient by the community pharmacist compared to the information provided by the physician.

The GFR (Glomerular Filtration Rate) value was ranked as the second most important patient data item to be made available for community pharmacists. This indicates the importance of kidney function in the implementation of individually optimized pharmacotherapies. Several medicines commonly used by older adults are eliminated through the kidneys and require dose adjustment based on reduced renal function to avoid adverse effects.^51^ Some medications should be even fully avoided due to impaired kidney function. The finding of this study is in line with previous studies showing the importance of kidney function as a patient data item to be routinely available in community pharmacies.^15^, ^18^, ^21^ A Japanese study by Yokoyama et al.^18^ found that kidney function was the most common test value leading community pharmacists to contact the hospital to inquire about the appropriateness of prescriptions. One potential way to make GFR values available to community pharmacists and other care team members is to include the most recent GFR value in electronic prescriptions, especially for people 65 years or older. This could ensure that kidney function is regularly considered while prescribing, dispensing, and following up medication use throughout the medication use process.

Other data items that the expert panel ranked as important to be available in community pharmacies for the statutory dispensing and counseling form a logical set of core items to be followed up for the patient's health status and medication self-management. For example, blood pressure, cholesterol, blood potassium, and glycosylated hemoglobin tests should be available because they are linked to the monitoring of many common chronic conditions such as cardiovascular diseases, diabetes, and metabolic syndrome, which require long-term commitment to treatment.^38^ Blood pressure monitoring is also important in circumstances other than essential hypertension, as many other conditions and medications can affect blood pressure.^52^, ^53^, ^54^ In addition, if community pharmacists had access to blood pressure monitoring data, they could better contribute to achieving treatment goals for hypertension, which has proven to be challenging.55., 56. The international normalized ratio (INR), hemoglobin, and drug plasma concentration values would allow community pharmacists to contribute to the monitoring of the therapeutic equilibrium of treatments with warfarin and other high-risk medications that more likely than other medicines can cause severe medication errors and harm to patients if inappropriately used.57., 58. This set of patient data items is also in line with a Dutch study^15^ that found data on renal function (GFR), electrolyte balance (potassium), blood coagulation (INR), blood glucose (glycosylated hemoglobin), and drug plasma concentration monitoring to be important in assessing and managing risks related to drug-drug interactions in community pharmacies.

A wider range of patient data should be available in community pharmacies for extended clinical pharmacy services than for the statutory dispensing and counseling, especially when conducting collaborative medication reviews. This is in line with the definitions of collaborative and comprehensive medication reviews that assess the appropriateness of the medication regimen in relation to a person's medical conditions.45., 46., 47, 48, 59., 60, 61 The patient data items ranked among the most important in this study were also identified as necessary for medication reviews in a German study by Greißing et al.^21^

### 4.1 Strengths and limitations of the study

The study utilized a panel of 20 experts, which is typical panel-size for this type of health service research applying the Delphi method.^39^, ^62^ Only clinical pharmacy specialists were included to ensure relevant experience, though this may limit the generalizability of the results to all community pharmacists, particularly those mainly involved in dispensing and counseling. Diversity was supported by selecting panelists from care teams outside community pharmacies. High engagement was maintained throughout, with response rates between 95% and 100%.

The Delphi method was chosen for its ability to build expert consensus in areas lacking empirical evidence, with iterative rounds that refine opinions and anonymous responses that reduce bias.^35^, ^36^, ^62^ However, it may still be affected by participant biases, groupthink, and limited generalizability due to reliance on a small panel. In this study, no formal coding or external validation for qualitative analysis occurred as the comments were primarily intended to guide revisions to the survey in later rounds. These factors should be considered when assessing the findings.

The Delphi rounds relied on literature and practical expertise to create a preliminary list of patient data items, which was pilot tested. The consensus threshold was set high, enhancing the study's reliability. Consensus on individual data items ranged from 0% to 100%, indicating that the experts were able to identify both necessary and less necessary data items. Clarifying the instructions in the second round helped align panelists' answers more closely with the intended clinical and prospective risk management perspectives of the study. However, this refinement may also have influenced panelists' behavior by increasing their awareness of how the questions were meant to be interpreted and therefore impacted the study's reliability. Between the first and the second round, one panelist who had not responded during the first round expressed willingness to participate in the following rounds. Participation was allowed as the second round was going to be very similar to the first one, although it is not the norm in Delphi studies. In the third round, only one “cannot say” response can be considered having only a minor impact on the final importance scores.

### 4.2 Policy and practice implications

This study is among the first to provide a more detailed and comprehensive understanding of the patient data that should be made available in community pharmacies. The findings offer important insights into health policy and strategic planning in community pharmacy practice and electronic health record (EHR) systems in Finland and beyond. They highlight the need to prioritize essential patient data items for community pharmacies. These results align with previous research from Japan,^18^ which recommended integrating laboratory test databases into dispensing processes. Direct access to patient data - without intermediaries - would improve efficiency and resource utilization. It would also strengthen pharmacists' roles in medication optimization, proactive risk management, and the development of advanced pharmacy services aligned with broader health and social care needs.

Access to patient data is critical for integrating community pharmacies into a closed-loop medication management process - a fully electronic system designed to ensure continuity of care and patient safety.^63^ Emerging digital tools and smart devices could further enable timely and reliable data access.1., 64. To manage medication risks effectively, pharmacies should actively participate in these systems and align with clinical pathways. Additionally, pharmacists need secure communication channels to share observations and medication review notes with other care team members.1., 8. Such collaboration would enhance patient care and support large-scale implementation of pharmacist-led medication reviews.^6^, ^7^, ^20^, ^22^, ^65^, ^66^

Implementing patient data access requires robust digital information management. In Finland, recent health and social service reforms have advanced digital care pathways and introduced the national Kanta medication list - a centralized record of prescriptions.5., 23., 67., 68. This will enable pharmacists to record medication reviews, as well as facilitate better information exchange between different healthcare actors. Making patient information visible in pharmacies is part of the development of the medication list. The results of this study provide valuable information to support this development work.

Panelists agreed that pharmacies should have access to substantial patient data but expressed concerns about its practical use. The main challenge lies in pharmacists' abilities to interpret clinical information and contribute to optimized pharmacotherapy. Competence levels vary widely, particularly in interpreting laboratory results and assessing drug-related risks. These concerns echo findings from previous studies.^33^, ^66^ Beyond data interpretation, panelists emphasized the need for understanding healthcare structures and processes to foster interprofessional, patient-centered collaboration. Therefore, additional training in applied pharmacotherapy and healthcare system familiarity is recommended, using methods such as case-based learning and participation in care teams.^66^

Since 2005, Finnish pharmacists have had voluntary opportunities for continuing education and in-house training to gain medication review expertise.45., 46., 47, 48 In 2017, medication review training was incorporated into the undergraduate curriculum, ensuring new graduates enter practice with stronger clinical skills.^47^, ^69^ Another option is to restrict data access to pharmacists with proven medication review expertise.

Panelists also raised concerns about time and financial constraints. In Finland, pharmacy income primarily comes from medicine sales, which cover mandatory counseling services. Pharmacies cannot charge extra for statutory counseling, and uptake of extended clinical services has been modest.^24^, 26., 31, 70 Future integration of clinical pharmacy services will depend on financial structures, incentives, and reimbursement models. For example, in Germany and other countries, national health insurance already funds clinical pharmacy services.^2^, ^71^

### 4.3 Future studies

This study reinforces the prevailing view that community pharmacies require access to a larger and more comprehensive set of patient data than is currently available. To validate these findings, pilot studies involving broader and more representative multiprofessional participants are recommended. Additionally, qualitative research should be conducted to provide deeper insights into the practical implications of data access. Future investigations should also examine pharmacists' competencies in interpreting patient data and assess the adequacy of resources within pharmacy settings.

Overall, sustained research and policy actions are needed to advance community pharmacists' roles in patient care and medication risk management.1., 8., 9. Post-implementation studies should evaluate how pharmacists utilize patient data and their impact on medication safety.

## 5 Conclusion

Consensus was reached for a relatively large set of patient data items to be available in community pharmacies, especially for providing clinical pharmacy services. Data on diagnoses, kidney function and blood pressure were rated most important. Open comments spontaneously revealed some concerns on pharmacotherapeutic expertise and resources in pharmacies for applying patient data to real-life practice. The findings are timely for guiding health policy making and strategic planning of community pharmacy practice and electronic health records in Finland and beyond.

## CRediT authorship contribution statement

**Johanna Laakso:** Writing – review & editing, Writing – original draft, Visualization, Project administration, Methodology, Investigation, Formal analysis, Data curation, Conceptualization. **Sonja Kallio:** Writing – review & editing, Supervision, Conceptualization. **Marja Airaksinen:** Writing – review & editing, Supervision, Methodology, Conceptualization. **Maarit Dimitrow:** Writing – review & editing, Supervision, Project administration, Methodology, Conceptualization.

## Funding

This research did not receive any specific grant from funding agencies in the public, commercial, or not-for-profit sectors. Open access funded by Helsinki University Library.

## Declaration of competing interest

The authors report no conflicts of interest.

## Data Availability

Inquiries regarding the data used in this study can be directed to the corresponding author (Johanna Laakso).
